# CT Findings of Patients with Small Bowel Obstruction due to Bezoar: A Descriptive Study

**DOI:** 10.1155/2013/298392

**Published:** 2013-04-18

**Authors:** Fatih Altintoprak, Bumin Degirmenci, Enis Dikicier, Guner Cakmak, Taner Kivilcim, Gokhan Akbulut, Osman Nuri Dilek, Yasemin Gunduz

**Affiliations:** ^1^Department of General Surgery, Faculty of Medicine, Sakarya University, Turkey; ^2^Department of Radiology, Faculty of Medicine, Sakarya University, Turkey; ^3^Department of Radiology, Faculty of Medicine, Suleyman Demirel University, Turkey; ^4^Department of General Surgery, Sakarya University Research and Educational Hospital, Turkey

## Abstract

*Purpose*. The aim of this study was to present the computed tomography (CT) findings of bezoars that cause obstruction in the small bowel and to emphasize that some CT findings can be considered specific to some bezoar types. *Materials and Methods*. The records of 39 patients who underwent preoperative abdominal CT and subsequent operation with a diagnosis of intestinal obstruction due to bezoars were retrospectively analyzed. *Results*. In total, 56 bezoars were surgically removed from 39 patients. Bezoars were most commonly located in the jejunum (*n* = 26/56, 46.4%). Sixteen (41.0%) patients had multiple bezoar locations in the gastrointestinal tract. Common CT findings in all patients were a mottled gas pattern and a focal ovoid or round intraluminal mass with regular margins and a heterogeneous internal structure. Furthermore, some CT findings were determined to be specific to bezoars caused by persimmons. *Conclusions*. Preoperative CT is valuable in patients admitted with signs of intestinal obstruction in geographic regions with a high bezoar prevalence. We believe that the correct diagnosis of bezoars and the identification of their number and location provide a great advantage for all physicians and surgeons. In addition, some types of bezoars have unique CT findings, and we believe that these findings may help to establish a diagnosis.

## 1. Introduction

Bezoar is a mass of swallowed foreign indigestible material found within the gastrointestinal tract (GIT). Despite the fact that bezoars are a rare cause of intestinal obstruction, this emergency pathology is a frequently encountered problem worldwide [1]. Predisposing factors in bezoar formation include systemic diseases that reduce gastrointestinal motility and previous peptic ulcer surgery [2]. 

Radiologic findings are very valuable for bezoar diagnosis, because clinical and laboratory findings are similar for bezoars and other causes. Radiographic and ultrasonographic findings have been defined for bezoars [3, 4]. However, both methods have disadvantages. Computed tomography (CT) is superior to other radiologic tools for bezoar diagnosis and differential diagnosis in patients with intestinal obstruction.

In this paper, we evaluated the preoperative CT findings of bezoars that cause obstructions in the small intestines, and highlight some special CT appearances that may be useful for differential diagnoses in the preoperative period.

## 2. Materials and Methods

The records of 39 patients who underwent preoperative abdominal CT and subsequent operation with a diagnosis of intestinal obstruction due to bezoars were retrospectively analyzed between January 2004 and December 2012 at single center. The diagnosis of intestinal obstruction was established on the basis of clinical presentation (vomiting and abdominal pain and/or distention) and radiologic findings (plain X-ray or CT). The diagnosis of intestinal bezoar was confirmed either by intraoperative findings or on CT findings.

All patients' CT examinations were performed with a 4-multidetector computed tomography (4-MDCT) scanner (Asteion; Toshiba, Kobe, Japan). The following parameters were used in the CT examination protocols: 4 × 5 mm collimation, 5 mm slice thickness, 2.5 mm scan interval, 120 kVp, and 250 mAs. Approximately 125 mL of intravenous (IV) iohexol (Omnipaque 300; GE Healthcare, Little Chalfont, United Kingdom), iopromide (Ultravist 300; Bayer-Schering, Berlin, Germany), or iomeprol (Iomeron 350; Bracco, Milano, Italy) was given to patients who have no contraindications for IV contrast use. Contrast-enhanced examinations using a routine portal venous phase (60–70 s) were performed. 

When considering the diameter and shape of bezoars, the axial CT sequences of bezoars which were located only in obstruction region were evaluated.

## 3. Results

The female : male ratio was 24 : 15 (1.6 : 1), and the mean age was 62.0 (range 28–82) years. Eighteen patients were >65 years of age. A total of 56 bezoars were found in 39 patients. 

Bezoars were most commonly located in the jejunum (*n* = 26/56, 46.4%). Other bezoar locations include ileum (*n* = 17/56, 30.3%), stomach (*n* = 12/56, 21.4%), and duodenum (*n* = 1/56, 1.7%). Sixteen (41.0%) patients had multiple bezoars in different GIT locations stomach and jejunum, one each in six patients (15.3%); stomach and ileum, one each in five patients (12.8%); jejunum and ileum, one each in two patients (5.1%); jejunum and stomach, two and one in one patient (2.5%); two bezoars in jejenum, two patients (5.1%) (Table 1). 

Twenty-nine (74.3%) patients had a history of previous abdominal surgery, twenty-one (21/29, 72.4%) of them due to peptic ulcers. The surgery had been performed an average of 13 (range 10–26) years previously. Nine of twenty-one (42.8%) patients who underwent previous abdominal surgery due to peptic ulcers had multiple bezoars. Six (15.3%) patients had diabetes mellitus (DM; type I, *n* = 2; type II, *n* = 4).

**Figure 1:**
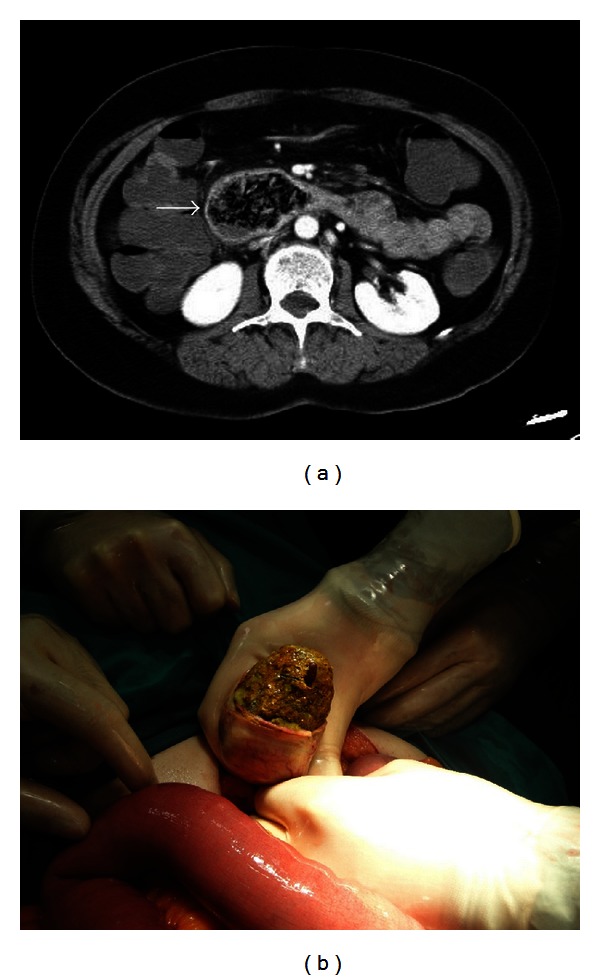
61-year-old female patient. (a) CT findings: an intraluminal ovoid bezoar. Fusiform-shaped seeds were seen in the mass, and wall thickening was seen in the duodenum (arrow). (b) Intraoperative findings: an ovoid-shaped bezoar containing seeds was removed via enterotomy.

**Figure 2:**
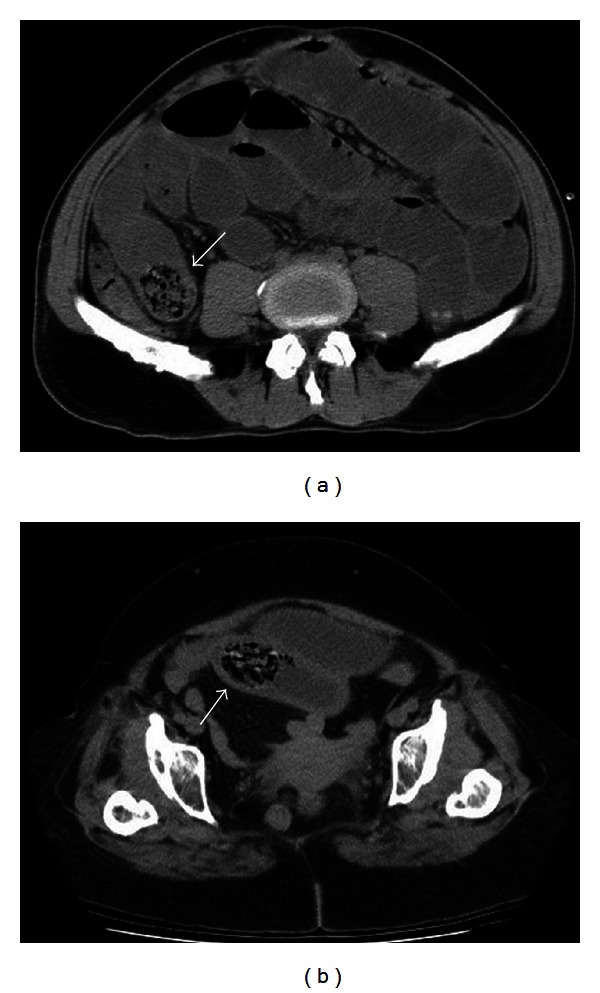
52-year-old female patient (a) and 62-year-old female patient (b) had an intraluminal round bezoar with a mottled gas pattern (arrows).

**Figure 3:**
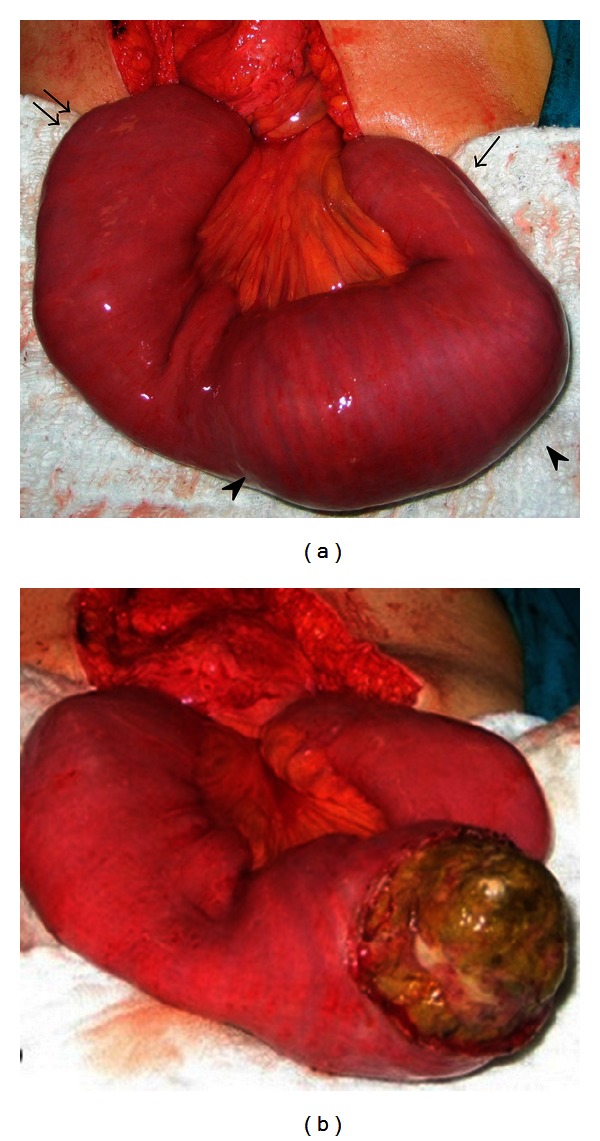
Intraoperative view of 62-year-old female patient. Intraluminally located bezoar (arrow heads), dilated proximal segments (double arrow), and nondilated distal segments (arrow) are visible.

Computed tomography findings of bezoars in the small intestine included a mottled gas pattern and a focal ovoid (*n* = 31/44, 70.4%; Figure 1) or round (*n* = 13/44, 29.6%) intraluminal mass located in the obstruction region with regular margins and a heterogeneous internal structure. In addition, while the small intestine proximal to the mass was dilated, the distal intestines had a normal diameter, suggesting intestinal obstruction (Figures 2 and 3). Contrast changes caused by inflammation at and proximal to the obstruction site were also detected. 

**Figure 4:**
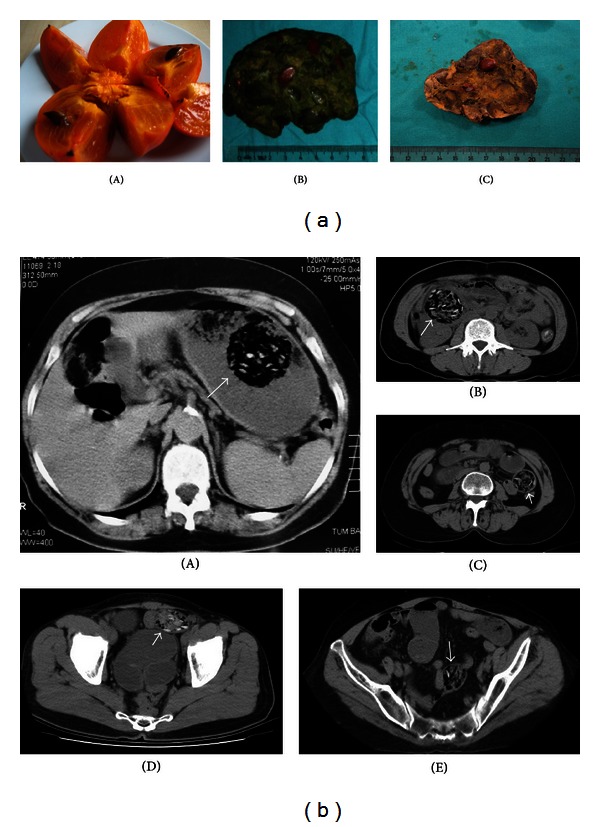
(a) Macroscopic appearance: (A) a seedy type of persimmon, (B)-(C) bezoars caused by a seedy type of persimmon. The bezoar seemed to contain a large number of seeds, and large air slits were seen. (b) CT findings of bezoars due to seedy type persimmon. Bezoar views which have different shapes and sizes and large amount of air and contained hyperdense ovoid seeds that had settled in different locations at different levels of the gastrointestinal system (arrows). (A) Stomach, (B)-(C) jejunum, and (D)-(E) ileum.

*Patients with bezoars due to seedy type persimmon *(*removed bezoars contained persimmon seeds*). Round or ovoid intraluminal masses with a heterogeneous internal structure and mottled gas pattern were detected at different levels of the small intestines and the stomach. As a result of our examines on CT images, we have also noticed that persimmons seeds (a) were seen as hyperdense structures within the bezoar, (b) showed a dispersed, rather than converging, seed distribution within the bezoar, and (c) contained greater amounts and wider intervals of air (Figures 4(a) and 4(b)).

## 4. Discussion

A 0.4% incidence of bezoars has been reported, but the exact rate is unknown [5]. Some factors, such as altered GIT anatomy (peptic ulcer operations), overconsumption of certain foods (e.g., persimmons), and the presence of diseases that affect GIT motility, are known to increase the possibility of bezoar formation [6]. The relationship between previous peptic ulcer operation (with resection or vagotomy and drainage procedures) and bezoar formation has been emphasized in the literature [2–4, 7]. In our series, 15.3% of patients had DM and 74.3% had a history of previous abdominal surgery. Furthermore, the majority (72.4%) of these abdominal operations were performed due to peptic ulcers.

Diagnosis of bezoars is difficult in the preoperative period. For this purpose, various imaging methods are used (abdominal graphy, barium studies, and ultrasonography), and specific disadvantages have been reported for each method [4, 8–10]. In our patients, we detected air-fluid levels on abdominal graphy suggestive only of intestinal obstruction, rather than a specific diagnosis. Moreover, in our clinic, we do not routinely perform barium X-rays and abdominal ultrasonography in patients with intestinal obstruction to obtain differential diagnoses.

CT is superior to other radiologic tools for diagnosis and differential diagnoses in patients with intestinal obstruction based on clinical and abdominal graphy findings. Preoperative CT examination can obtain very valuable information about the level of obstruction, etiology, and presence of additional pathology, and can facilitate the planning of the operation type [11, 12]. When evaluating CT images, a general knowledge about bezoars is required, including which bezoars (approximately one-third) may be synchronous [13]. Hoover et al. [14] emphasized this important detail in a report of a patient who required a second operation because of an overlooked bezoar, despite preoperative CT examination. In our series, sixteen (41.0%) patients had multiple bezoars, and nine patients of them (56.2%) had a history of previous abdominal surgery due to peptic ulcers. The most frequent location of multiple bezoars was the jejunum, and all synchronous bezoars were identified on preoperative CT examination.

General and pathognomonic CT findings of bezoars have been described in the literature. Additionally, it has been established that bezoar diagnosis can be made by CT in the preoperative period [9, 10, 15–22]. In our patients, we have accurately determined number and locations of all bezoars by preoperative CT examination.

The mentioned determinations about bezoars due to persimmon overconsumption (contained greater amounts and wider intervals of air, exhibited hyperdense seeds, and showed a dispersed, rather than converging, seed distribution within the bezoar) are subjective and may not be unique to persimmon seeds. But these findings may be typical features for this specific type of bezoar, and we believe that they should be kept in mind when evaluating abdominal CT findings in some geographic regions. 

The appearances about small bowel bezoars may be similar to small bowel feces (SBF). SBF is frequently found within the lumen of a relatively long segment of a dilated small bowel loop, and the incidence is approximately 8% in small bowel obstruction cases [15]. SBF is placed proximal to the obstruction site as distinct from bezoar and longer than bezoar. Kim et al. [13] defined that SBF is more tubular shaped than bezoar, and Zissin et al. [15] indicated that the length of the feces-like material found within the dilated loop proximal to the transition zone is the key for differentiating SBF sign from a bezoar. We did not think SBF diagnosis in our cases due to the fact that the intraluminal gas-containing mass imagings with round or ovoid-shaped bezoar are located at the obstruction site. 

Carrying out only the axial section CT findings' evaluation is the restrictive aspect of our study. By the multidetector row CT (MDCT), more detailed assessments can be made by taking axial, coronal, sagittal, and oblique images in several sequences. Hodel et al. [23] found that multiplanar reformations (MPRs) can increase both accuracy and confidence in the location of the transition zone in CT of mechanical small bowel obstruction. If our study was designed by MPR, probably different results could be achieved (especially about shape of the bezoar at the obstruction region).

In conclusion, although bezoars are a rare cause of intestinal obstruction, the possible presence of bezoars should not be forgotten in patients admitted with signs of intestinal obstruction, especially patients with a history of previous abdominal surgery for peptic ulcer. In addition, we believe that some bezoars, especially those caused by persimmons, have specific CT appearances that may help to establish a preoperative diagnosis in some patients admitted with signs of intestinal obstruction and who live in geographic regions where persimmon overconsumption is common.

## Figures and Tables

**Table 1:** Total bezoars and multiple bezoar locations.

Total bezoar locations	*n* = 56, (100%)	Multiple bezoars locations	*n* = 39, (100%)
Stomach	12 (21.4%)	Stomach and jejunum	6 (15.3%)
Duodenum	1 (1.7%)	Stomach and Ileum	5 (12.8%)
Jejunum	26 (46.4%)	Jejenum and ileum	2 (5.1%)
Ileum	17 (30.3%)	^ #^Stomach and jejenum	1 (2.5%)
		*Jejenum	2 (5.1%)

^#^One bezoar in stomach and two bezoars in jejunum.

*Two bezoars in jejenum.
